# Editorial: Molecular and breeding mechanisms for enhanced performance in underutilized leguminous crops in Africa

**DOI:** 10.3389/fgene.2024.1385989

**Published:** 2024-03-05

**Authors:** Lydia N. Horn, M. T. Labuschagne

**Affiliations:** ^1^ Multidisciplinary Research Services, University of Namibia, Windhoek, Namibia; ^2^ Department of Plant Sciences, University of the Free State, Bloemfontein, South Africa

**Keywords:** legumes, molecular breeding, food security, Africa, underutilized crops

Legumes such as chickpea (*Cicer arietinum*), common bean (*Phaseolus vulgaris*), Bambara groundnut (*Vigna subterranea*), cowpea (*Vigna unguiculata*), groundnut (*Arachis hypogaea*), pigeon pea (*Cajanus cajan*), and soybean (*Glycine max*) are some of the most crucial food security crops in Africa, although they are generally underutilized. Despite their importance for being nutritious and drought-tolerant, field performance and productivity of these crops remain low, and they have been poorly researched. Limited genetic improvement or breeding activities have been reported for these crops. Therefore, this Research Topic focused on documenting the approaches deployed to identify useful traits such as adaptive features, high-yielding traits, breeding methods, and the role of emerging technologies such as mutation breeding using various technologies, sequencing, and high-throughput phenotyping to increase the utilization of important genetic resources in Africa’s pre-breeding programs.

Four papers were published within this topic. The first “A comprehensive investigation of lipid-transfer proteins from Cicer arietinum disentangles their role in plant defense against Helicoverpa armigera*-infestation*” by Saxena et al., was on Lipid Transfer Proteins (LTPs) which are very important in synthesizing lipid barrier polymers and are involved in defense signaling during pest and pathogen attacks. The study was done on *C. arietinum* (chickpea) where 48 LTPs were identified and structurally and functionally characterized. This data highlighted the role of LTPs in various stress responses and plant developmental processes and contributed to knowledge on the genetics, evolution and distribution of these proteins in chickpeas.

The second paper “Gamma irradiation-induced genetic variability and its effects on the phenotypic and agronomic traits of groundnut (*A. hypogaea* L.)” by Saibari et al., confirmed that groundnut is a major oilseed crop which is very important for human and animal nutrition as it has high protein and oil content. Two Moroccan groundnut genotypes with an erect habit and short growing cycle were treated with various doses of cobalt-60 (60Co) gamma-ray (Gy) to induce mutagenesis. Gy generated novel agronomic and phenotypic variants with the highest number of mutants identified at 100 and 150 Gy. They determined the LD50 from the radiosensitivity test, which will benefit future mutagenic research. This genetic gain can be stabilized in segregating generations, and the genome of desirable mutants can be used to develop and increase the variability of groundnut.

The next paper “Metabolome profile variations in common bean (Phaseolus vulgaris L.) resistant and susceptible genotypes incited by rust (Uromyces appendiculatus)” by Makhumbila et al. reiterated that rust is a major constraint for common bean (Phaseolus vulgaris) production as it causes substantial yield losses in many common bean production areas worldwide. *A*lthough there have been numerous breakthroughs in resistance breeding for this disease, the pathogen remains a threat as it has been shown to mutate. In this study, metabolome profiles of a common resistant and a common susceptible cultivar were investigated for their response to U. appendiculatus races (1 and 3) at 14- and 21-days post-infection using liquid chromatography-quadrupole time-of-flight tandem mass spectrometry. Non-targeted metabolomics identified 71 known metabolites that were putatively annotated, of which 33 were statistically significant. Rust infection in both genotypes caused the expression of key metabolites such as flavonoids, terpenoids, alkaloids and lipids. They concluded that a timely response to pathogen attack by signaling the production of specific metabolites can be used as a strategy to understand plant defense.

The last paper “The Pea R2R3-MYB Gene Family and Its Role in Anthocyanin Biosynthesis in Flowers” by Yang et al. confirms that Pea (*Pisum sativum* L.) is an important legume crop with excellent nutritional value. Crop metabolic engineering is increasingly being applied to improve the color, flavor and nutritional value of pea. The ternary MYB–bHLH–WD repeat protein (MBW) complex regulates the anthocyanin biosynthesis pathway, and research has been targeted on the members of the *R2R3-MYB* gene family to improve the valuable metabolic product of crops. They identified 119 *R2R3-MYB* genes in the assembled pea genome, of which 111 were distributed across 14 chromosomes. By combining these with 126 R2R3-MYB protein sequences of *Arabidopsis*, they categorized 245 R2R3-MYB proteins into 36 subgroups according to sequence similarity and phylogenetic relationships. This study provides a good reference to further characterize the diverse functions of R2R3-MYB genes and helps researchers understand the color formation of pea flowers.

This Research Topic therefore covered a wide range of areas related to legume genetics, and covered plant defense systems, mutation breeding and metabolomics as tools to better understand the genetics of legumes and how it can be applied in the genetic improvement of these crops.

